# Detection of *Plasmodium falciparum* male and female gametocytes and determination of parasite sex ratio in human endemic populations by novel, cheap and robust RTqPCR assays

**DOI:** 10.1186/s12936-017-2118-z

**Published:** 2017-11-17

**Authors:** Federica Santolamazza, Pamela Avellino, Giulia Siciliano, Franck Adama Yao, Fabrizio Lombardo, Jean Bosco Ouédraogo, David Modiano, Pietro Alano, Valentina Dianora Mangano

**Affiliations:** 1 Dipartimento di Sanità Pubblica e Malattie Infettive, Sapienza Università di Roma, Roma, Italy; 2 Dipartimento di Malattie Infettive, Istituto Superiore di Sanità, Roma, Italy; 3 Institut de Recherche en Sciences de la Santé, Bobo Dioulasso, Burkina Faso; 4 Centre Muraz, Bobo Dioulasso, Burkina Faso; 5 Dipartimento di Ricerca Traslazionale e Nuove Tecnologie in Medicina e Chirurgia, Università di Pisa, Pisa, Italy

**Keywords:** Malaria transmission, *Plasmodium falciparum*, Gametocytes, Sex-ratio, Real Time qPCR

## Abstract

**Background:**

The presence of *Plasmodium falciparum* gametocytes in peripheral blood is essential for human to mosquito parasite transmission. The detection of submicroscopic infections with gametocytes and the estimation of the gametocyte sex ratio are crucial to assess the human host potential ability to infect mosquitoes and transmit malaria parasites.

**Aim and objectives:**

The aim of this work was to develop sensitive and cheap Real Time qPCR assays for large-scale epidemiological surveys, based on detection and amplification of gametocyte sex specific transcripts selected from the literature: the female-specific *pfs25* and *pf glycerol kinase* (*pfGK*) and the male-specific *pfs230p* and *pf13* transcripts.

**Methods:**

RTqPCR assays were used to test the gametocyte- and sex-specific expression of the target genes using asexual stages of the gametocyteless parasite clone F12 and FACS purified male and female gametocytes of the PfDynGFP/P47mCherry line. Assays were performed on 50 blood samples collected during an epidemiological survey in the Soumousso village, Burkina Faso, West-Africa, and amplification of the human housekeeping gene *18S rRNA* was employed to normalize RNA sample variability.

**Results:**

SYBR Green assays were developed that showed higher sensitivity compared to Taqman assays at a reduced cost. RTqPCR results confirmed that expression of *pfs25* and *pfs230p* are female and male-specific, respectively, and introduced two novel markers, the female-specific *pfGK* and the male-specific *pf13*. A formula was derived to calculate the ratio of male to female gametocytes based on the ratio of male to female transcript copy number. Use of these assays in the field samples showed, as expected, a higher sensitivity of RTqPCR compared to microscopy. Importantly, similar values of gametocyte sex-ratio were obtained in the field samples based on the four different target combinations.

**Conclusion:**

Novel, sensitive, cheap and robust molecular assays were developed for the detection and quantification of female and male *P. falciparum* gametocytes. In particular, the RTqPCR assays based on the female-specific *pfs25* and the newly described male gametocyte-specific *pf13* transcripts, including normalization by the human *18S,* reliably assess presence and abundance of female and male gametocytes and enable to determine their sex-ratio in human subjects in endemic areas.

**Electronic supplementary material:**

The online version of this article (10.1186/s12936-017-2118-z) contains supplementary material, which is available to authorized users.

## Background

Despite a remarkable decrease in mortality and morbidity over the last 10 years thanks to the successful application of control measures, malaria still imposes a major public health burden: in 2015 malaria caused 212 million clinical cases and 429,000 deaths worldwide, 90% of which occurred in sub-Saharan Africa, where *Plasmodium falciparum* is the most prevalent species [1].

Transmission of malaria parasites from the human host to the mosquito vector requires the presence of mature gametocytes in the peripheral blood. Mature (stage V) gametocytes are morphologically and physiologically distinguishable as males or females and circulate in peripheral blood for an average of 4.6–6.5 days [2–4]. Parasite transmission to the vector is a very efficient process as it has been shown that mosquitoes can be infected even when feeding on subjects carrying sub-microscopic gametocyte densities, corresponding to < 4 gametocytes/μl [5, 6]. As the infection to the mosquito requires the mating of two gametes of opposite sex, the gametocyte sex ratio, i.e. the proportion of gametocytes that are male, is a critical parameter to evaluate/predict infectiousness. In malaria parasites, gametocyte sex ratio is female biased, can vary during the course of individual infections and is affected by parasite density and transmission level [7, 8].

Highly sensitive molecular tools relying on mRNA amplification, such as Reverse Transcriptase quantitative PCR (RTqPCR) and Quantitative Nucleic Acid Sequence Based Amplification (qNASBA), have been developed to detect gametocytes in peripheral blood targeting sexual stage-specific transcripts [9, 10]. The higher sensitivity of these assays compared to microscopy has been consistently described in the last years [11–14].

However, existing assays pose important cost constraints for use in the field as they are based on quite expensive qPCR Taqman and qNASBA technologies. Furthermore, they do not include an internal control gene, making them susceptible to variation in RNA quantity and quality among samples. Finally, the significant variability between reports on the absolute number of gametocyte-specific transcript copies corresponding to one gametocyte prevents to consistently translate the assay readouts into actual numbers of gametocytes.

The aim of this work was to develop sensitive, cheap and robust RTqPCR assays based on amplification of sex specific transcripts for use in large-scale epidemiological surveys. Based on previously published assays [15] and on a recent transcriptomic analysis of male and female gametocytes [16], four target genes were selected: the female-specific *pfs25* and *pfGK*, and the male-specific *pfs230p* and *pf13* transcripts. SYBR Green assays were developed for the detection of the above transcripts that showed higher sensitivity and reduced costs compared to existing Taqman assays, and introduced amplification of a reference gene (the human *18S rRNA*) to normalize transcript copy number in field samples. Finally, a protocol to derive gametocyte sex ratio from the ratio of the male to female transcript copy number was described and tested on a panel of blood samples collected during an epidemiological survey in the Soumousso village, Burkina Faso, West-Africa.

## Methods

### Parasite cultures

The *P. falciparum* lines 3D7A [17], DynGFP/P47mCherry [16] and F12 [18] were cultured in human 0+ erythrocytes at 5% haematocrit under 5% CO_2_, 2% O_2_, 93% N_2_ [19]. Cultures were grown in RPMI 1640 medium (Gibco) supplemented with 25 mM Hepes, 50 µg/ml hypoxanthine, 0.25 mM NaHCO_3_, 50 µg/ml gentamicin sulfate, 10% pooled heat-inactivated O+ human serum. The PfDynGFP/P47mCherry line was cultured under selection of 5 µg/ml blasticidin.

### Gametocyte culture and purification

Parasites from the 3D7A and the DynGFP/P47mCherry lines were induced to produce gametocytes by parasite overgrowth. After a 5-day treatment with 50 mM *N*-acetyl-glucosamine (NAG) to eliminate residual asexual parasites, stage III/IV gametocytes were partially purified from uninfected erythrocytes on MACS Separation Columns CS (Miltenyi Biotec). Gametocytes were then put back in culture and allowed to mature to stage V over the following 7 days. Aliquots of 5 × 10^5^ mature gametocytes were centrifuged and pellets were stored at − 80 °C for further analyses.

### Isolation of trophozoites and ring stages from the F12 line

Asynchronous parasites from the *P. falciparum* F12 line were grown to high parasitaemia (8%). Trophozoites were purified from uninfected erythrocytes and from ring stage parasites over a 60% Percoll cushion [20], counted in a haemocytometer chamber and aliquots were centrifuged and resuspended in 50 µl of medium and then in 450 µl of RNAlater (Applied Biosystem). Other aliquots of purified trophozoites were further grown at a low haematocrit (2%) for additional 26 h to obtain synchronous, newly invaded ring stage parasites. Aliquots of ring stage parasites were centrifuged and resuspended in 50 µl of medium and then in 450 µl of RNAlater.

### Culture and purification of male and female gametocytes of the PfDynGFP/P47mCherry line

Parasites from the PfDynGFP/P47mCherry line were induced to produce gametocytes as above and stage III/IV gametocytes were partially purified from uninfected erythrocytes on MACS Separation Columns CS (Miltenyi Biotec). Gametocytes were resuspended (10 × 10^6^/ml) in 1× PBS and male and female gametocytes were separated by sorting at room temperature using a BD FACSAria flow cytometer (Becton–Dickinson). First, gametocytes were separated from uninfected erythrocytes using forward and sideward scatter and then the GFP+ (male) gametocytes were separated from the GFP− (female) gametocytes. Aliquots of the sorted samples were analysed by UV fluorescence microscopy and Giemsa stained to determine the purity of the gametocyte populations. The GFP+ and GFP− gametocytes were put back in separate cultures and were allowed to mature to stage V over the following 7 days. Mature gametocytes were counted in a haemocytometer chamber and aliquots of male and female gametocytes were centrifuged and resuspended in 50 µl medium and then in 450 µl of RNAlater.

### Epidemiological survey and study sample

An epidemiological survey was conducted during the peak of the 2013 malaria high transmission season (July–September) in the Soumousso village (11°00′46″N, 4°02′45″W), Burkina Faso, West Africa. Whole blood samples were collected from 500 subjects aged from 2 to 20 years of both sexes in order to investigate the *P. falciparum* infection reservoir in the population and how it varies with host factors (manuscript in preparation). Blood was collected by finger-prick in a 1.5 ml tube containing EDTA and 50 µl were immediately added to 450 µl of RNALater solution and stored at − 20 °C. Venous blood from Italian healthy donors (N = 3) were collected and a pool was used as negative control.

A representative sample subset from 50 subjects, belonging to different age groups and of both sexes, was selected from the original collection and used for the present work using cluster random sampling according to microscope positivity for *P. falciparum* gametocytes. Random sampling was used to select 14 (1/3) samples from subjects microscopy-negative for *P. falciparum* gametocytes and 36 (2/3) samples from subjects microscopy positive for *P. falciparum* gametocytes.

### Microscopic examination

Thick and thin blood smears were prepared according to the WHO guidelines for the microscopic diagnosis of malaria [21], for the detection and quantification of *P. falciparum* gametocytes density by light microscopy (LM). Gametocyte density was estimated per microlitre of blood, by reading 100 microscopic fields (~ 20 leukocytes per field at × 1000 corresponding to ~ 0.25 μl of blood) of the thick blood smear. Microscopic readings were performed in duplicate by two experienced microscopists and the mean value of the reading was used in the data analysis.

### RNA extraction and cDNA synthesis

500 μl blood/RNA later (50 μl blood, 450 μl RNALater) were centrifuged and resuspended in 250 μl of DEPC-treated water. RNA extraction was then performed using TRIzol (Life Technologies) reagent following the manufacturer’s protocol and the RNA pellet was finally resuspended in 20 μl DEPC-treated water. Quality and concentration of isolated RNA was evaluated using the microplate spectrophotometer Take3 (Synergy HT, BIOTEK). RNA samples were treated with TURBO™ DNase (Ambion) to remove contaminant genomic DNA and cDNA synthesis was performed using the High Capacity cDNA Reverse Transcription kit (Life Technologies), according to the manufacturer’s instruction. The efficacy of DNAse treatment was tested for each sample by RT minus PCR.

### SYBR Green assay primers design

Primers (*pfs25, pfGK, pf13, pf230p,* Table 1) for SYBR Green assays were designed using the Primer Express software (Applied Biosystems) following recommended guidelines for qPCR primer design. Primers do not span introns, and gDNA contamination was avoided as described above. In order to prevent non-*P. falciparum*-specific amplification, primers were checked for homology by performing BLASTn analysis against genomes of *Plasmodium* species infecting humans other than *P. falciparum* (PlasmoDB, Orthology and Synteny tool), as well as against the human genome (NCBI). To experimentally check the specificity of each primer pair a melting curve analysis was added at the end of the PCR cycles. Each primer pair produced a single amplicon and showed a clean amplification as revealed by single sharp peaks (Additional file 1: Figure S1).

**Table 1:** Selected *P. falciparum* gene targets and primers of SYBRGreen RTqPCR assays

Gene ID	Gene alias	Chromosome	Forward primer	Reverse primer
PF3D7_1031000	*pfs25*	10	TGGAAATCCCGTTTCATACGC	ACCGTTACCACAAGTTACATTCT
PF3D7_1351600	*pfGK*	13	AAGTTGTATATTCCACATGCGGTT	TATGCACCCAGATGGAGATCTGATG
PF3D7_1311100	*pf13*	13	AGAACGAATATGCTCGAGAACGAATTAGAAA	TGCCTTTTCATCTGACACGT
PF3D7_0209000	*pfs230p*	2	CCCAACTAATCGAAGGGATGAA (Schneider et al. [15])	TGTTGTTCGATTCCAGTTGGT

The table shows the gene ID, gene alias, chromosome, forward and reverse primer sequences for amplification of female (*pfs25 and pfGK*) and male (*pf13* and *pfs230p*) gametocyte-specific *P. falciparum* transcripts

### Standard curves of target constructs

Genomic DNA was extracted from a culture of *P. falciparum* using the QIAamp DNA Mini Kit (Qiagen) following manufacturer’s instruction. DNA amplicons of each *P. falciparum* gametocyte target (*pfs25, pfGK, pf13, pf230p*) and of the human reference gene (*18S rRNA*) were produced by end point PCR, using specific primers tailed with T7 promoter sequences to enable in vitro RNA transcription (Additional file 1: Table S2). Total RNA was therefore transcribed using the T7 RNA Polymerase Kit (Promega). After DNAse treatment, the concentration of RNA was determined by spectrophotometer analysis and copy numbers were estimated using ENDMEMO tool (http://www.endmemo.com/bio/dnacopynum.php). Finally, cDNA was prepared as described above and tenfold dilutions were used to obtain standard curves using three replicates per dilution. The amplification efficiency (E) of the qPCR assays is estimated on the basis of the equation E = (10 − 1/slope − 1) × 100. R^2^ is the coefficient of correlation obtained for the standard curve and should be > 0.99. The Limit of Quantification (LOQ) is the lowest concentration of construct (copies/µl) that can be quantified by a given qPCR assay.

### qPCR

The qPCR reactions were performed using a StepOnePlus Real-Time PCR system (Applied Biosystems). Each SYBR Green qPCR reaction (final volume of 20 μl) contained 10 μl of 2× Master mix SYBR Green (Applied Biosystems), 225 nM of Forward and Reverse primers and 2 μl of cDNA. The thermal cycle conditions were as follows: 10 min at 95 °C, followed by 40 cycles at 95 °C for 15 s, 60 °C for 1 min. A melting curve analysis was added at the end of each reaction, with fluorescence detection every 0.2 °C. *pfs25* Taqman protocol was performed following the protocol described by Schneider et al. [15]. All samples were analyzed in duplicate. In each plate, a negative control (uninfected sample), a negative amplification control and a standard curve for each target were included. A Ct value of < 40 was used as threshold of positivity. Absolute quantification protocol was applied to evaluate *P. falciparum* target genes copy numbers, using the standard curves of the target constructs previously described. Relative quantification was used to assess male and female gametocyte numbers, using standard curves generated from serial dilutions of male and female gametocytes isolated from cultures as described above. *P. falciparum* target genes copy numbers extrapolated for field samples were further normalised using the endogenous control gene by expressing them as a ratio to the to the concentration of the h18S transcript (ng/μl) measured in the same qPCR experiment.

### Statistical analyses and graphs

Statistical analysis and graphics were performed using GraphPad Prism 6.0 (GraphPad Software, Inc. La Jolla, CA). Nonlinear standard curve interpolations were applied to fit data from RTqPCR experiments: Cq values from marker’s absolute quantifications were correlated to Cq values from purified female or male gametocytes relative quantifications. Nonlinear regression analysis was also employed to fit data from field samples in a straight line in log–log graphs (ratio of Male/Female gametocytes on Y-axis, ratio of copies of Male Marker/Female Marker on X-axis). Slope, intercept and R^2^ parameters were calculated for each equation. Resulting equation formulas were used to interpolate ratios of Male/Female gametocytes from RTqPCR values (copies of Male/Female Markers) in human blood samples using all four primers combinations.

Absolute (counts) and relative (percentages) frequencies of gametocyte-positive samples in the field collection were calculated and showed in bar plots using Microsoft Office Excel 2007.

Linear correlation between transcript copy number/μl and gametocytes/μl in field samples was assessed by correlation coefficient (r^2^) and Spearman test (rho, *p* value) using STATA v.10 (StataCorp. 2007. Stata Statistical Software: Release 10. College Station, TX: StataCorp LP.).

## Results and discussion

### Selection of target and reference genes

The *P. falciparum pfs25* (PF3D7_1031000) and *pf230p* (PF3D7_0209000) genes were selected respectively as markers of female and male gametocytes in RTqPCR assays according to Schneider et al. [15]. The *P. falciparum pfGK* gene (PF3D7_1351600) was initially selected to detect and quantify both male and female gametocytes, based on the evidence that this gene is both gametocyte-specific and expressed at very high levels [22, 23]. Since the work by Lasonder et al. showed that *pfGK* is a female-specific transcript (Additional file 1: Table S1), as it turned out to be the case for virtually all highly expressed gametocyte-specific transcripts, *pfGK* was used as a further female-specific assay. As the analysis of Lasonder et al. [16] showed that *pf230p* mRNA is not a top ranking male-specific transcript, a new male gametocyte-specific marker was developed using gene *pf13* (PF3D7_1311100), as in that analysis this mRNA ranked first as male gametocyte-specificity, with the lowest female to male expression ratio (Additional file 1: Table S1).

To overcome the inherent sample variability in RNA quantity and quality expected in field samples, a human reference gene was selected to normalize gametocyte- and sex-specific target quantification. To choose a reference gene evenly expressed across the samples, qPCR was analysed for four human genes: *18S rRNA* [24], *B2M* (β-2-microglobulin) [25], *ACTB* (β-actin) [26], *HPRT* (hypoxanthine phosphoribosyl-transferase) [27]. The four genes were tested in 20 of the 50 cDNA samples collected in the epidemiological survey in the Soumousso village, from males and females of different age groups. According to the uniformity of expression *18S rRNA* has been chosen as reference gene.

### Analytical validation of the assays

Specific parameters (melting temperatures, standard curve slope and efficiency) relative to PCR amplification for each primer pair were evaluated and resulted satisfactory for downstream application (Additional file 1: Figure S1 and S2, Table S3). The LOQ, i.e. the lowest concentration of construct (copies/reaction) that can be quantified with acceptable precision and accuracy, is equal to 100 copies/μl for *pfs25*-Taqman, 10 copies/μl for *pfs25*-SYBR Green, 100 copies/μl for *pfGK*, 73.6 copies/μl for *pf13*, and 100 copies/μl for *pf230*p (Additional file 1: Table S2).

### Stage and sex specific expression of target genes

Stage-specific expression of the selected target genes was assessed performing RTqPCR assays on asexual ring stages and trophozoites of the F12 *P. falciparum* strain and from gametocytes of the PfDynGFP/P47mCherry strain. The F12 line was used because it is unable to produce morphologically recognisable gametocytes [18, 28] as it is mutated in the AP2-G transcription factor, a master regulator of gametocyte differentiation [29]; these parasites were used as source of asexual stages to minimize the possibility of amplifying sexual stage transcripts from the low level of gametocyte contamination virtually unavoidable in asexual stage samples prepared from wild type parasite lines.

**Fig. 1:**
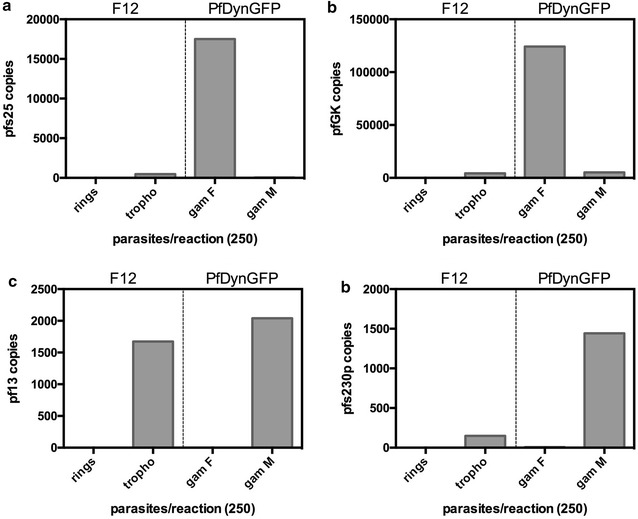
Gametocyte-specific expression of target genes. Copy number of the indicated transcripts measured in asexual (rings, trophozoites) and sexual (female and male gametocytes) stages of the F12 and PfDynGFP parasite lines, respectively

The *pfs25* and *pfGK* female markers showed a high level of expression in the gametocyte stage, a very low level of expression in trophozoites and an undetectable level of expression in ring stages (Fig. 1). The *pf13* and *pf230p* male markers showed comparable expression levels in gametocytes and in trophozoites, but no detectable level of expression in ring stage parasites (Fig. 1). These results indicate that the expression of female gametocyte markers *pfs25* and *pfGK* is highly specific for the sexual stages. In contrast, the male gametocyte markers *pf13* and *pfs230p* could be amplified also from asexual trophozoites. This indicates that the latter assays are not suited to quantify male gametocytes in in vitro cultures of asynchronous sexual and asexual blood stages. However, importantly, these assays can be confidently used with a high specificity for the sexual stages in field blood samples, as ring stages are the only asexual parasites found in circulation in peripheral blood.

**Fig. 2:**
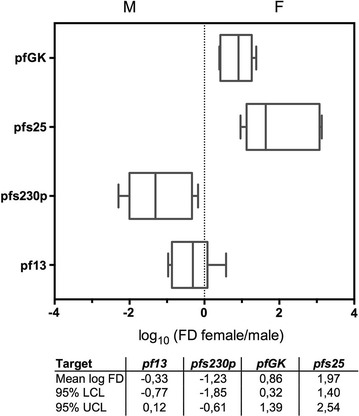
Sex-specific expression of target genes. Box and whiskers plot reporting data from assays on different sorted gametocyte populations. Two independent female/male sex-specific sorting by FACS procedure (see “Methods”) were used to test each target gene (the graph reports data from 8, 9, 5 and 12 replicates for pf13, pfs230p, pfGK and pfs25 respectively). Copies number for each target were obtained as described in the text, setting established putative amount of female/male gametocyte as reference. For each target, number of copies calculated in the female sorted population were divided with number of copies calculated in the male sorted population. The fold difference of expression (FD) obtained was converted to logarithmic scale for plotting purposes

The sex-specific expression of the target genes was assessed by RTqPCR on samples of purified female and male gametocytes, which were obtained by FACS sorting of GFP+ fluorescent male gametocytes and GFP− female gametocytes of the *P. falciparum* line PfDynGFP/P47mCherry [16]. Sorted sub-populations of female and male gametocytes were counted and used as templates to evaluate the expression intensity of each target. Two independent sorting experiments were considered. Differences in copy number of each target gene between the two sorting experiments were observed (Additional file 1: Figure S3), likely due to variability accumulated between samples in the experimental steps from cell sorting to cDNA production. However, when transcript copy numbers were used to calculate for each target the fold difference of expression (FD) in female gametocytes compared to males, the female/male FD values were consistent across biological and technical replicates (Fig. 2). These results therefore confirmed the expected sex-specificity of all target genes both at high and low gametocyte density (250 gam/reaction; 25 gam/reaction; 2.5 gam/reaction): female markers *pfs25* and *pfGK* are strongly enriched in the female gametocytes samples (logFD_pfs25_ = 1.97 and logFD_pfGK_ = 0.86), whereas male markers *pf13* and *pfs230p* are strongly enriched in the male gametocytes samples (logFD_pf13_ = − 0.33 and logFD_pfs230p_ = − 1.23).

### Estimation of gametocyte sex ratio

**Fig. 3:**
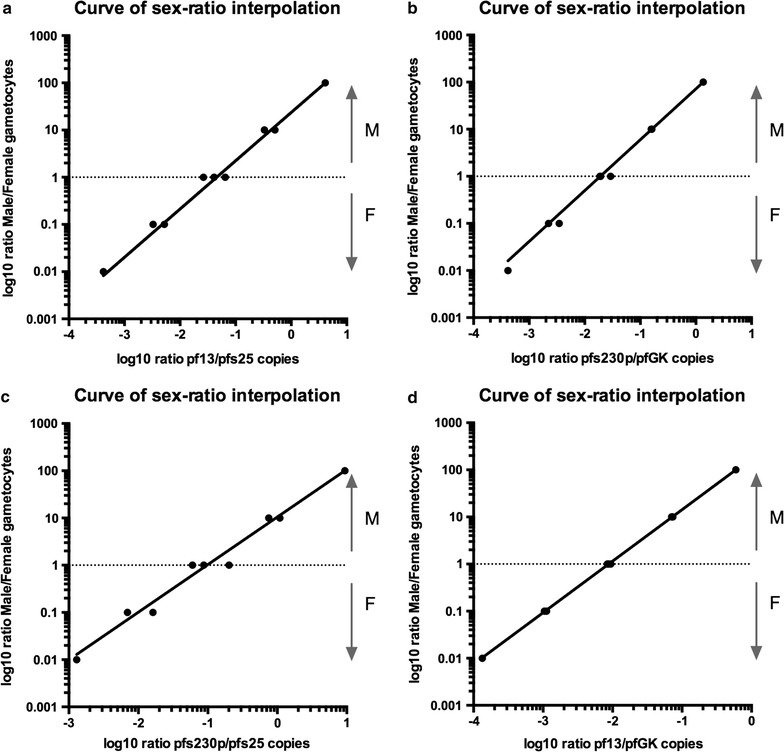
Correlation curves between the ratio of male to female transcript copies and the ratio of male to female gametocytes. For each combination of targets, the figure shows the correlation curve between the ratio of male to female mean transcript copy number (x axis) and the ratio of male to female gametocyte (y axis). The data used to build the curves are shown in Additional file 1: Figure S3. The mean transcript copy number was obtained for each target by averaging the number of copies obtained from replicates of sorting experiments. Gametocytes sex ratios were as follows: 0.01 (2.5M/250F), 0.1 (25M/250F, 2.5M/25F), 1 (2.5M/2. 5F, 25M/25F, 250M/250F), 10 (25M/2.5F, 250M/25F), 100 (250M/2.5F). The following formula was used to extrapolate gametocyte sex ratio from RTqPCR data for mixed cultures and field samples: ${\text{Female/male}}\;{\text{gametocyte}}\;{\text{ratio}}\,{ = }\, 1 0^{{\, ( {\text{slope}}\,\, \times \,{ \log }\, ( {\text{n}}\,{\text{male}}\,{\kern 1pt} {\text{transcript}}\,\,{\text{copies/n}}\,\,{\text{female}}\,\,{\text{transcript}}\,\,{\text{copies)}}\,{ + }\,{\text{Y}}\, \times \,\,{\text{intercept)}}}}$. Slope and intercept for the different target combinations are shown in Additional file 1: Table S4

Determination of gametocyte sex ratio (male/female) is a useful tool to evaluate *Plasmodium* infectivity. Here, a method is proposed to extrapolate gametocyte sex ratio in a blood sample based on the number of male and female transcript copies quantified by RTqPCR. Results of assays performed on two independent purifications of female and male gametocytes of the *P. falciparum* line PfDynGFP/P47mCherry were used to build a linear correlation between the ratio of male to female transcripts copy number and the ratio of male to female gametocytes. Figure 3 shows the correlation curves for each targets pair. The slope and intercept of a curve can be used to estimate the gametocyte sex ratio in field samples (see below) using the formula.

$${\text{Gametocyte}}\,{\text{sex}}\,{\text{ratio}}\,{ }= 1 0^{{\, ( {\text{slope}}\;{ \times}\;{ \log }\,( {\text{n}}\,{\text{copies}}\,{\text{female}}\,{\text{marker/n}}\,{\text{copies}}\,{\text{male}}\,{\text{marker)}}\;{ + }\,{\text{Y}}\,{ \times}\,{\text{intercept)}}}}$$

Using this formula researchers can obtain gametocyte sex-ratios by inputing their own RTqPCR data.

### Performance of RTqPCR assays on blood samples collected in the field

A pilot test of the performance of the proposed protocol, including RTqPCR of the four gametocyte targets and the human reference gene, was conducted on 50 RNA samples extracted from whole peripheral blood collected during an epidemiological survey in Soumousso, Burkina Faso. The results obtained by RTqPCR assays were compared with those of microscopy, which identified gametocyte presence in 34 samples out of 50. The *pfs25* assay was performed with both Taqman and SYBR Green technologies to compare performance of the two methods.

**Fig. 4:**
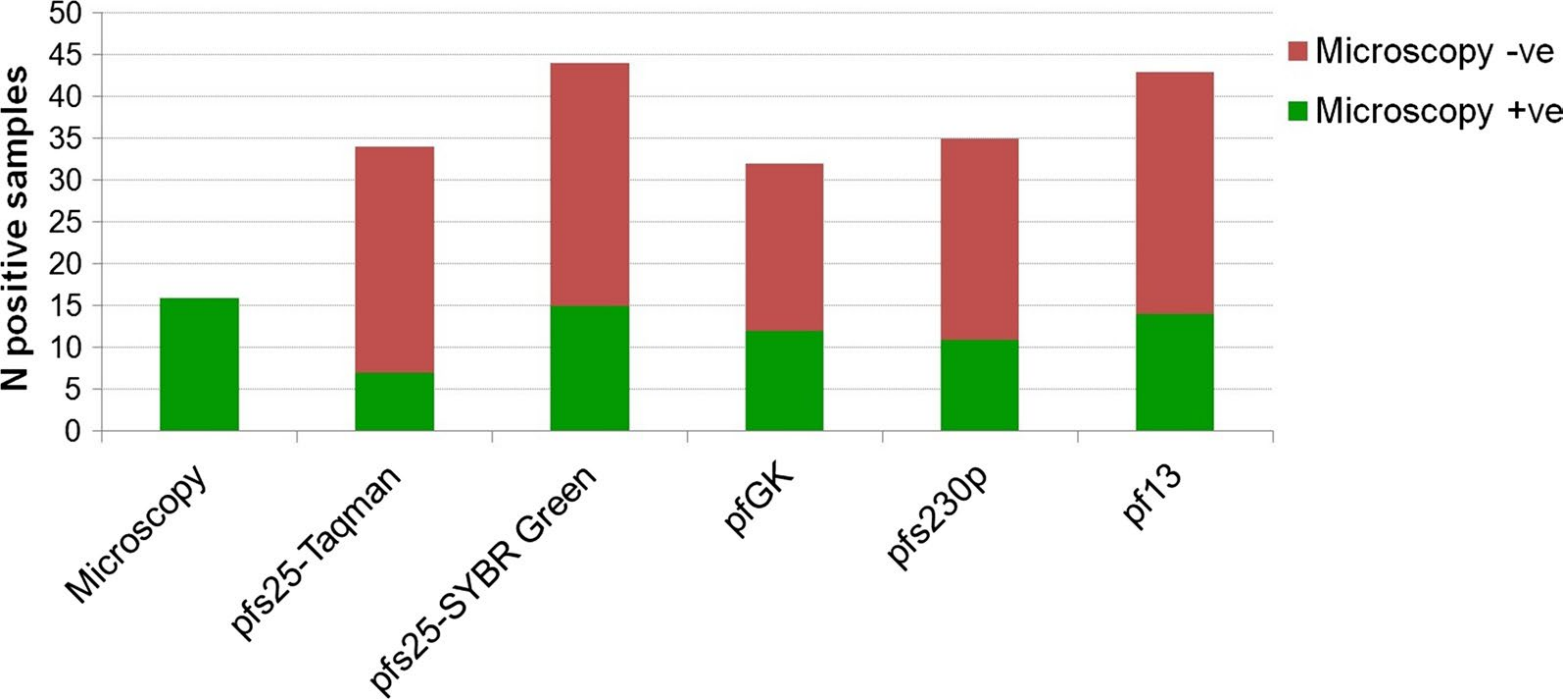
Number of samples identified as *P. falciparum* gametocyte positive by microscopy and by RTqPCR assays. The figure shows the number of samples identified as *P. falciparum* gametocyte positive by microscopy and by RTqPCR assays. For qPCR assays, the number of positive samples is expressed as a sum of microscopy positive and microscopy negative samples

As expected, all gametocyte assays identified a higher number of gametocyte-positive samples compared to microscopic examination (Fig. 4). Among female gametocyte markers, the *pfs25*-SYBR Green assay identified the highest number of positive samples (N = 44, 88%), 15 out of 16 microscopy positive samples (94%), and 29 out of 34 negative samples (85%). Among male gametocyte markers, the *pf13* assay identified the highest number of positive samples (N = 43), 14 out of 16 microscopy positive samples (88%), and 29 out of 34 negative samples (85%). In the majority of the above samples (n = 37, 74%) gametocytes were detected by both assays. Interestingly, in one case a microscopy-positive sample was negative for both *pfs25* (SYBR Green and Taqman) and *pfGK* assays but positive for both the *pfs230p* and *pf13* assays, raising the intriguing possibility that this infection contained in that moment a vast excess of male gametocytes. Similarly, in one of the two instances where the *pf13* assay was negative among the microscopy positive samples, this sample was also negative for *pfs230p* while it was positive for both *pfs25* and *pfGK* assays, suggesting that this infection contained only female gametocytes.

In *pfs25* assays, SYBR Green resulted in a higher number of positive samples (N = 44), compared to Taqman (N = 34, of which 32 or 94% also positive by SYBR Green). The lower sensitivity of *pfs25*-Taqman with respect to *pfs25*-SYBR Green in this experiment could be at least partly explained by a poorer performance of the Taqman assay when RNA quantity/quality is low (i.e. h18S quantity less than 1 ng/μl, Additional file 1: Figure S4).

**Fig. 5:**
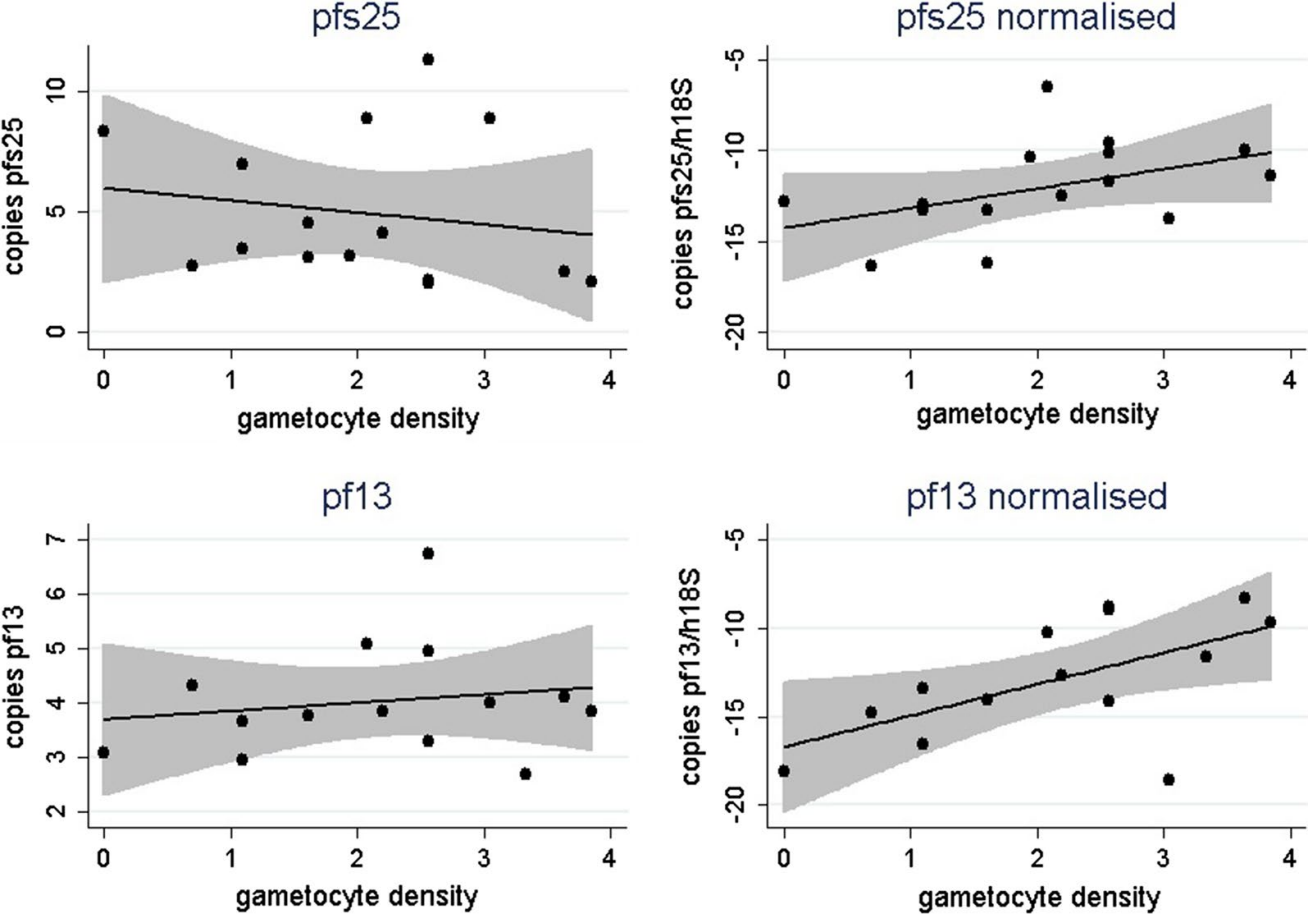
Correlation between the number of gametocytes and the number of *P. falciparum* gametocyte transcript copies, before and after data normalization by human 18S. The Figure shows the linear correlation between the number of gametocytes per μl (log 10 gametocyte density, x axis) and the number of *P. falciparum* gametocyte transcript copies per μl (log10 copies, y axis), before (left panel: *pfs25* and *pf13*) and after (right panel: *pfs25* normalised and *pf13* normalised) data normalisation by human 18S

The impact of RNA quality/quantity and normalization by human 18S rRNA was further assessed by measuring the correlation between the number of gametocytes/μl as assessed by microscopy and the number of gametocyte transcript copies/μl assessed by RTqPCR, in absence and presence of normalization. For *pfs25*, linear correlation coefficient increased from r^2^ = 0.031 prior to normalization (Spearman rho = − 0.278; p = 0.315) to r^2^ = 0.200 after normalization (Spearman rho = 0.522; p = 0.046); similarly, for *pf13*, linear correlation coefficient increased from r^2^ = 0.028 prior to normalization (Spearman rho = 0.155; p = 0.598) to r^2^ = 0.356 after normalization (Spearman rho = 0.568; p = 0.034) (Fig. 5).

**Fig. 6:**
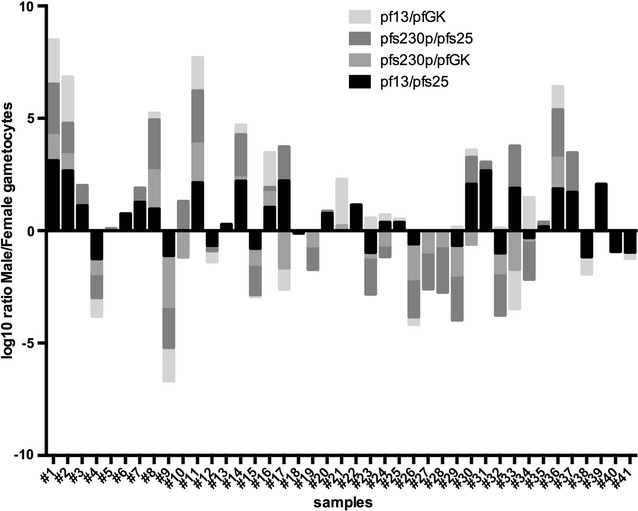
Sex ratio values determined by different male–female target pairs. The figure shows the value of the male/female gametocyte ratio calculated in field samples using RTqPCR data for the four different combination of male and female markers

Finally, the formulas described above were applied to derive gametocyte sex-ratio in each field sample from RTqPCR data, using the four combination of male and female specific transcripts. Results obtained using different target combinations were very similar (Fig. 6).

### Analysis of costs

In order for researchers to make a cost-informed decision as to the protocols of choice for their studies, cost per sample for each major step of the work were compiled: (i) collection of blood from survey participant in the field (including EDTA tubes, RNAlater, tips and tubes) amounted to 0.60€ per sample; (ii) obtaining cDNA from blood (RNA extraction, DNAse treatment, cDNA synthesis) amounted to 3.10€ per sample; (iii) RTqPCR of *P. falciparum* gametocyte targets and human reference target cost 0.55€ per assay per sample using SYBR Green technology, and 1.35€ per sample per assay using Taqman technology. The total cost per sample to obtain both pfs25 and pf13 data normalised for h18S using SYBR Green assays is 5.35€, with a reduction of 0.80€ per sample compared to using the pfs25-Taqman assay.

## Conclusions

The aim of this work was to develop molecular, RNA-based assays to detect female and male *P. falciparum* gametocytes at sub-microscopic densities and to estimate gametocyte sex ratio in human blood samples. Based on the recent literature, *pfs25* and *pfGK* were selected as female gametocyte markers and *pf13* and *pfs230p* as male gametocyte markers. Sensitive (LOQ ≤ 100 copies/μl) RTqPCR assays were developed for all markers using SYBR Green technology, which results in a substantial reduction of costs compared to previously published protocols based on Taqman or qNASBA. The gametocyte stage specificity of expression was confirmed for all markers, as well as the female-sex specificity of expression for *pfs25* and *pfGK* and the male-sex specificity of expression for *pf13* and *pfs230p*. A robust correlation was observed between the ratio of male to female transcript copy number and the ratio of male to female gametocytes, and derived formulas to calculate gametocyte sex ratio in a blood sample based on RTqPCR data. The gametocyte assays, along with a human reference gene assay for data normalization, were tested in blood samples collected during a malaria epidemiological survey in the village of Soumousso, Burkina Faso. All assays were able to detect a higher number of gametocyte positive samples compared to microscopy, as expected, with those for *pfs25* and *pf13* as the most sensitive assays. The *pfs25* SYBR Green assay was more sensitive and less affected by RNA availability compared to the *pfs25* Taqman assay. Normalization with the human reference gene enabled to establish a positive correlation of transcript copy number with microscopy gametocyte counts. Gametocyte sex-ratio based on different male and female marker pairs showed consistent results in field samples. These assays can therefore be used in epidemiological studies and in clinical trials on transmission blocking interventions to detect *P. falciparum* sub-microscopic densities of male and female gametocytes and to estimate gametocyte sex-ratio in human infected blood. These are crucial parameters to assess the infectivity of individuals to mosquitoes and to investigate factors affecting malaria transmission at population level.

## Additional file

**Additional file 1: Table S1.** *Plasmodium falciparum* genes showing the 10 lowest female to male mRNA ratio (male specific) and the 10 highest female to male mRNA ratio (female specific), from Lasonder et al. [16]. **Table S2.** Primer sequences for amplification of target constructs. **Table S3**. Limit of Quantification and quality parameters of RTqPCR assays. **Table S4.** Parameters of the formula to extrapolate gametocyte sex ratio from RTqPCR data. **Figure S1.** Melting curves of RTqPCR assays. **Figure S2.** Standard Curves of RTqPCR assays. **Figure S3.** Expression of target genes in male and female *P. falciparum* gametocytes. **Figure S4.** Sensitivity of *pfs25* Taqman and *pfs25* SYBR Green assays according to RNA quality/quantity.
