# Supplementary material for: Detection of Plasmodium falciparum male and female gametocytes and determination of parasite sex ratio in human endemic populations by novel, cheap and robust RTqPCR assays
Source: Malar J. 2017 Nov 17;16:468. doi: 10.1186/s12936-017-2118-z (PMC5693539; doi:10.1186/s12936-017-2118-z)
Supplement: Supplementary file 1 — Additional file 1: Table S1. Plasmodium falciparum genes showing the 10 lowest female to male mRNA ratio (male specific) and the 10 highest female to male mRNA ratio (female specific), from Lasonder et al. [16]. Table S2. Primer sequences for amplification of target constructs. Table S3. Limit of Quantification and quality parameters of RTqPCR assays. Table S4. Parameters of the formula to extrapolate gametocyte sex ratio from RTqPCR data. Figure S1. Melting curves of RTqPCR assays. Figure S2. Standard Curves of RTqPCR assays. Figure S3. Expression of target genes in male and female P. falciparum gametocytes. Figure S4. Sensitivity of pfs25 Taqman and pfs25 SYBR Green assays according to RNA quality/quantity. [file 12936_2017_2118_MOESM1_ESM.doc]

**ADDITIONAL MATERIAL**

**Additional Table S1. *P. falciparum* genes showing the 10 lowest female to male mRNA ratio (male specific) and the 10 highest female to male mRNA ratio (female specific), from Lasonder *et al.* 2016.**

| **Gene ID** | **Data from Lasonder et al. 2016** | | |  | **Selected for the present study** |
| --- | --- | --- | --- | --- | --- |
|  | **MG** | **FG** | **FG/MG ratio** | **Sex specificity** |  |
| PF3D7_1311100 | 2548.67 | 12.59 | 0.00 | male | *pf13* |
| PF3D7_1201600 | 17813.52 | 236.63 | 0.01 | male |  |
| PF3D7_1469900 | 10110.85 | 208.93 | 0.02 | male |  |
| PF3D7_0422300 | 3618.96 | 89.36 | 0.02 | male |  |
| PF3D7_1325200 | 738.25 | 23.20 | 0.03 | male |  |
| PF3D7_0717500 | 761.76 | 29.24 | 0.04 | male |  |
| PF3D7_0205000 | 933.69 | 38.04 | 0.04 | male |  |
| PF3D7_1444800 | 986.82 | 76.87 | 0.08 | male |  |
| PF3D7_0928100 | 1402.43 | 127.08 | 0.09 | male |  |
| PF3D7_1413200 | 3859.07 | 362.29 | 0.09 | male |  |
| PF3D7_1214500 | 19.46 | 971.41 | 49.91 | female |  |
| PF3D7_1362600 | 32.56 | 1474.74 | 45.29 | female |  |
| PF3D7_1454900 | 27.14 | 1228.48 | 45.27 | female |  |
| PF3D7_0825800 | 33.52 | 1451.28 | 43.30 | female |  |
| PF3D7_1351600 | 19.97 | 826.88 | 41.41 | female | *pfGK* |
| PF3D7_1146100 | 41.47 | 1683.22 | 40.58 | female |  |
| PF3D7_0827400 | 35.55 | 1311.66 | 36.89 | female |  |
| PF3D7_0630200 | 94.06 | 3351.21 | 35.63 | female |  |
| PF3D7_1031000 | 206.36 | 7341.64 | 35.58 | female | *pfs25* |
| PF3D7_0518800 | 48.14 | 1633.18 | 33.92 | female |  |

For each gene, the Table shows the Gene ID, female gametocyte mRNA level (FG), male gametocyte mRNA level (MG), female to male mRNA ratio (FG/MG ratio), sex-specificity of expression, whether the gene was selected for assay development in the present work. It is noteworthy that *pf230p* is not included in the list.

**Additional Table S2. Primer sequences for amplification of target constructs.**

| **Gene ID** | **Gene Alias** | **Forward Primer** | **Reverse Primer** |
| --- | --- | --- | --- |
| PF3D7_1031000 | *pfs25* | T7 –TCTGAAATGTGACGAAAAG  (Schneider et al. 2015) | TAACAGGATTGCTTGTATC |
| PF3D7_1351600 | *pfGK* | T7- AAGTTGTATATTCCACATGCGGTT | TATGCACCCAGATGGAGATCTGATG |
| PF3D7_1311100 | *pf13* | T7- TTATGAACGTACATCAGGAGAAGAT | ATTTGGTAAGAGGCACCGAT |
| PF3D7_0209000 | *pfs230p* | T7- ATGAGAAAACACAATGAACGTTCT | GTTCCTCATCAACATATTCCCTCTT |
| *Homo sapiens* 100008588 | *h18S* | T7- ttagagggacaagtggcgtt | TAGTCAAGTTCGACCGTCT |

The table shows, for each gene target, the forward and reverse primers used to amplify constructs used to build qRT-PCR standard curves.

**Additional Table S3. Limit of Quantification and quality parameters of RTqPCR assays.**

| **Target** | **LOQ copies/μl** | **Slope** | **E (%)** | **R^2^** |
| --- | --- | --- | --- | --- |
| *pfs25* Taqman | 100 | -3.36 | 98.23 | 0.99 |
| *pfs25* SYBR Green | 10 | -3.08 | 110.92 | 0.99 |
| *pfGK* | 100 | -3.12 | 108 | 0.98 |
| *pf230p* | 100 | -3.67 | 88.34 | 0.99 |
| *pf13* | 73.6 | -3.32 | 100.14 | 0.99 |
| *h18S* | 1000 | -3.11 | 109.7 | 0.99 |

The table shows for each target: limit of quantification (LOQ), slope, efficiency (E), and the coefficient of reproducibility (R^2^). The amplification efficiency (E) of the qPCR assays is estimated on the basis of the equation *E = (10−1/slope − 1) × 100.* R^2^ is the coefficient of correlation obtained for the standard curve and should be >0.99. The Limit Of Quantification (LOQ) is the lowest concentration of construct (copies/µl) that can be quantified by a given qPCR assay.

**Additional Table S4. Parameters of the formula to extrapolate gametocyte sex ratio from RTqPCR data.**

The table shows slope and intercept parameters for the different target combinations.

| **Target combination** | **Slope** | **Intercept** | **R^2^** |
| --- | --- | --- | --- |
| *pf13*/*pfs25* | 1.023 | 1.372 | 0.999 |
| *pfs230p*/*pfGK* | 1.080 | 1.857 | 1.000 |
| *pfs230p*/*pfs25* | 1.008 | 1.021 | 0.999 |
| *pf13*/*pfGK* | 1.093 | 2.246 | 1.000 |

The table shows slope and intercept parameters for the different target combinations, and the R^2^ value obtained from linear correlation curves.

**Additional Figure S1. Melting curves of RTqPCR assays.**


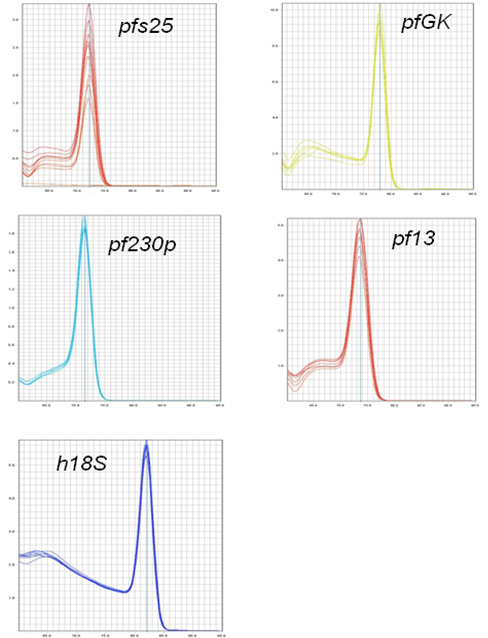


The figure shows the melt curves for five different amplicons, respectively *pfs25, pfGK, pf230p, pf13, h18S*. The single peak observed for all the targets is typically interpreted as representing a pure, single amplicon.

**Additional Figure S2. Standard Curves of RTqPCR assays.**

**
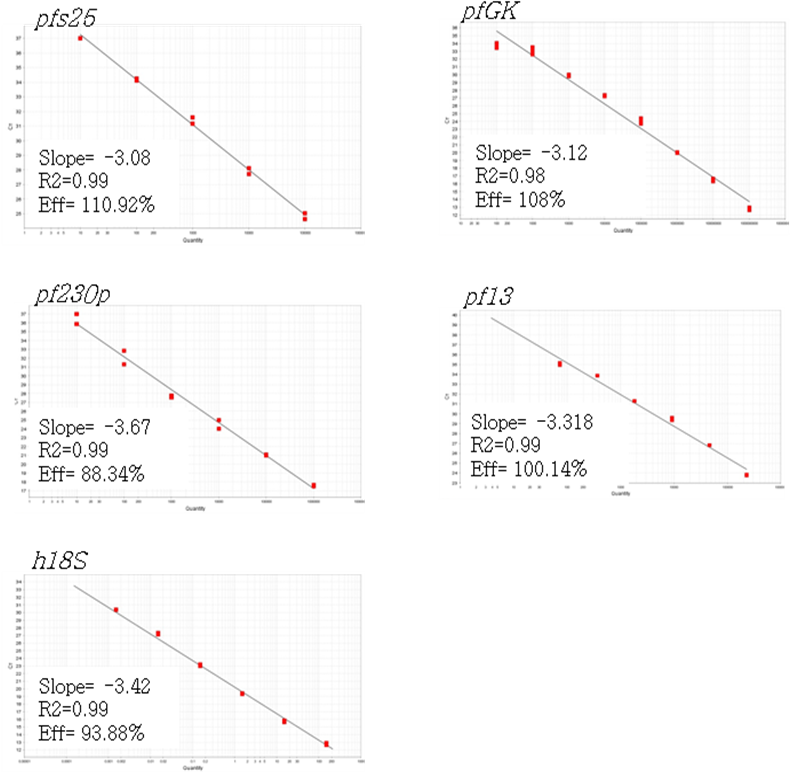
**

The Figure shows standard curves obtained using 10-fold dilutions of the construct prepared for the different targets; respectively, five points starting from 10^5^ copies/μl for *pfs 25*, eight points starting from 10^9^ copies/μl for *pfGK*, six points starting from 10^6^ copies/μl *pf230p*, six points starting from 7,3*10^6^ copies/μl for *pf13* and six points starting from 10^8^ copies/μl for the reference gene *h18S*.

**Additional Figure S3. Expression of target genes in male and female *P. falciparum* gametocytes.**

**
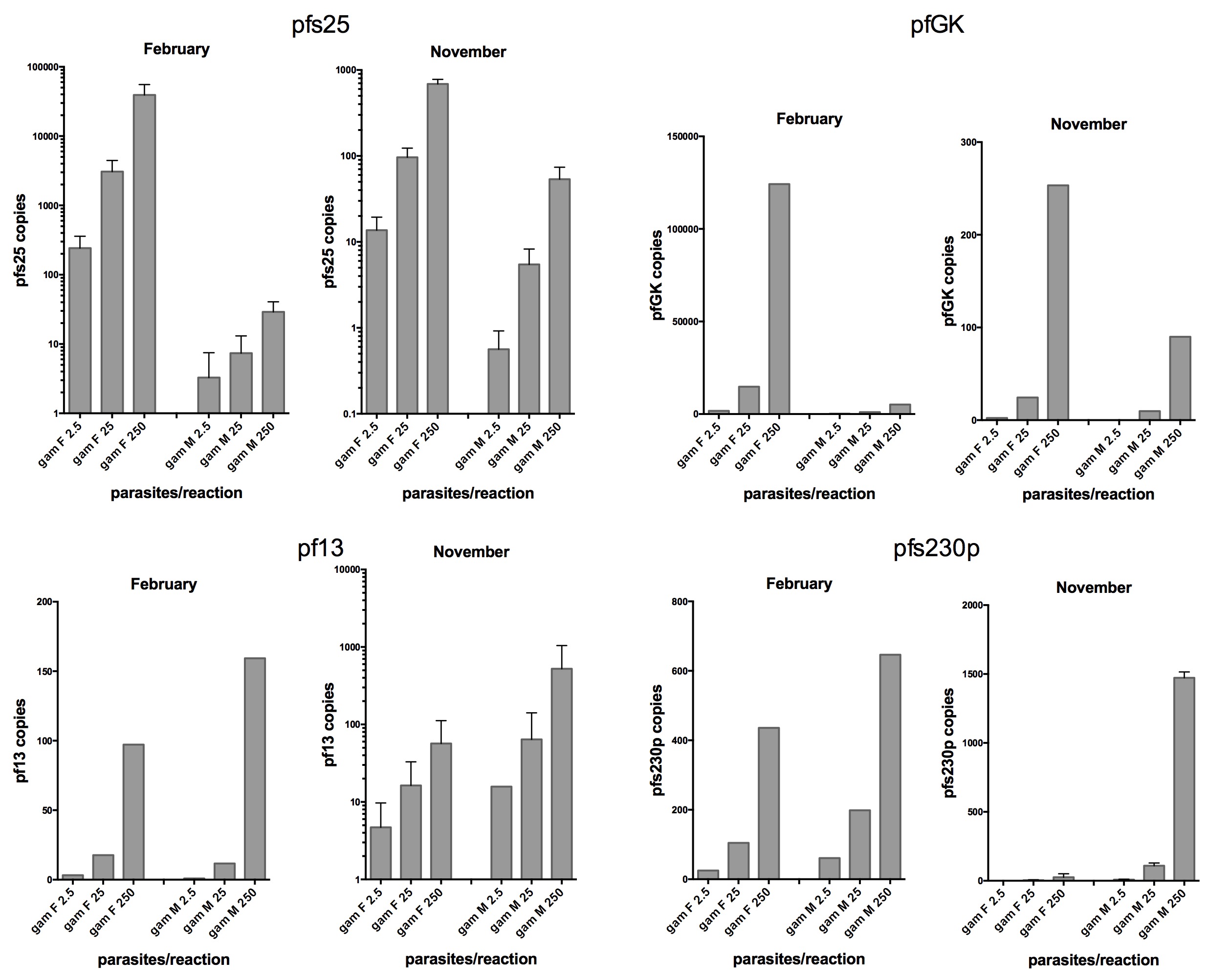
**

Real Time reactions were performed using in each plate a standard curve of gene construct dilutions to correlate Ct values with target genes copy numbers. We have set three gametocyte amounts (2.5, 25 and 250 gametocyte/reaction) to evaluate the correlation between parasite numbers and gene copies in different density conditions. We have therefore obtained corresponding Ct values and then copy numbers were extrapolated. In this Figure are summarized the results obtained considering two biological replicates (N, sorting experiments conducted in November 2016 and February 2017) and technical replicates (n: *pfs25*, two replicates for each sorted sample; *pf13* and *pfs230p*, two replicates for one sorting, one replicate for the other one; *pfGK*, one replicate for each sorted sample).

We observed significant differences in absolute copy numbers of each target gene between the two sortings, likely due to variability accumulated between samples in the experimental steps from cell sorting to cDNA production. The tables below show the mean transcript copy number of female and male markers, obtained by averaging results of biological and technical replicates of female and male gametocytes, respectively. These data have been used to build the correlation curves between male to female transcript copies and male to female gametocytes (Figure 3).

| **Female markers** | | |
| --- | --- | --- |
| Gene | pfs25 | pfGK |
| N | 2 | 2 |
| n | 2, 2 | 1, 2 |
| mean 2.5 FG | 127.9 | 882.7 |
| mean 25 FG | 1588.4 | 7412.3 |
| mean 250 FG | 19949.5 | 62244.7 |

| **Male markers** | | |
| --- | --- | --- |
| Gene | pf13 | pf230 |
| N | 2 | 2 |
| n | 1, 1 | 1, 2 |
| mean 2.5 MG | 8.3 | 25.8 |
| mean 25 MG | 65.1 | 139.0 |
| mean 250 MG | 525.3 | 1197.3 |

**Additional** **Figure S4. Sensitivity of *pfs25* Taqman and *pfs25* SYBR Green assays according to RNA quality/quantity.**


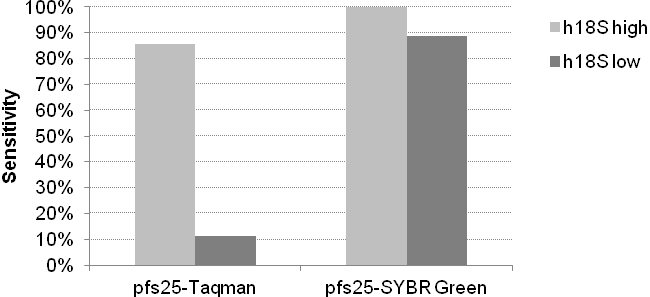


The Figure shows the sensitivity (% of microscopy positive samples identified as positive by RTqPCR) of *pfs25* Taqman and *pfs25* SYBR Green assays according to h18S quantity (high: h18S quantity >1ng/μl; low : h18S quantity ≤1ng/μl) as a marker of RNA quality/quantity in the qPCR reaction.
